# Cardiovascular Risk in Psoriasis: A Population-Based Analysis with Assessment of the Framingham Risk Score

**DOI:** 10.1155/2013/371569

**Published:** 2013-01-31

**Authors:** Elena Myasoedova, Bharath Manu Akkara Veetil, Eric L. Matteson, Hilal Maradit Kremers, Marian T. McEvoy, Cynthia S. Crowson

**Affiliations:** ^1^Division of Rheumatology, Mayo Clinic College of Medicine, 200 First Street SW, Rochester, MN 55905, USA; ^2^Department of Health Sciences Research, Mayo Clinic College of Medicine, 200 First Street SW, Rochester, MN 55905, USA; ^3^Department of Dermatology, Mayo Clinic College of Medicine, 200 First Street SW, Rochester, MN 55905, USA

## Abstract

*Objective*. To examine the utility of the Framingham risk score (FRS) in estimating cardiovascular risk in psoriasis. *Methods*. We compared the predicted 10-year risk of cardiovascular events, namely, cardiovascular death, myocardial infarction, heart failure, percutaneous transluminal coronary angioplasty, and coronary artery bypass grafting using the FRS, to the observed risk of cardiovascular events in a population-based cohort of patients with psoriasis. Patients with incident or prevalent adult-onset psoriasis aged 30–79 years without prior history of cardiovascular disease were included. *Results*. Among the 1197 patients with predicted risk scores, the median FRS was 6.0%, while the observed 10-year cardiovascular risk was 6.9% (standardized incidence ratio (SIR): 1.14; 95% confidence interval (CI): 0.92–1.42). The SIR was not elevated for women nor for men. The differences between observed and predicted cardiovascular risks in patients <60 years (SIR: 1.01; 95% CI: 0.73–1.41) or ≥60 years (SIR: 1.26; 95% CI: 0.95–1.68) were not statistically significant. *Conclusion*. There was no apparent difference between observed and predicted cardiovascular risks in patients with psoriasis in our study. FRS reasonably estimated cardiovascular risk in both men and women as well as in younger and older psoriasis patients, suggesting that FRS can be used in risk stratification in psoriasis without further adjustment.

## 1. Introduction

There is increasing interest in cardiovascular implications of chronic inflammatory conditions such as psoriasis [1]. Epidemiologic observations have demonstrated increased rates of cardiovascular events in patients with psoriasis [2, 3]. Some studies suggested that psoriasis itself may be an independent cardiovascular risk factor [4, 5], while others did not support this finding [6]. It is unclear whether cardiovascular risk in psoriasis is increased beyond that conferred by traditional cardiovascular risk factors and whether traditional risk assessment tools such as the Framingham risk score (FRS) are useful for cardiovascular risk stratification in psoriasis. We aimed to examine the utility of the FRS in estimating cardiovascular risk in psoriasis.

## 2. Materials and Methods

This retrospective population-based study was conducted using the Rochester epidemiology project (REP), a medical records linkage system containing complete inpatient and outpatient records from all healthcare providers in Olmsted County, Minnesota. The unique features of the REP were described previously [7]. The study protocol was approved by the Institutional Review Boards of Mayo Clinic and Olmsted Medical Center.

Using the REP resources, we identified all Olmsted County, Minnesota, residents aged 30–79 years with diagnostic codes consistent with psoriasis (International Classification of Diseases, Ninth Revision (ICD-9) codes 696.1, 696.2, 696.8, 696.5, and 696.3) between 01-01-1998 and 01-01-2008 [8]. Psoriasis was validated by a confirmatory diagnosis of dermatologist or a physician's description of the lesions in the medical record or a skin biopsy. Doubtful diagnoses were resolved by the dermatologist coinvestigator. The study included patients with incident or prevalent adult-onset psoriasis in 1998–2007. The psoriasis incidence date of incident subjects and the prevalence date for prevalent subjects (i.e., first mention of psoriasis during the study period) are further referred to as the baseline.

The entire inpatient and outpatient medical records of each patient were reviewed by trained nurse abstractors, beginning with the subjects' records at age 18 years or date of migration to Olmsted County until the death, migration, or end of the study (12-31-2010). Data were collected on cardiovascular events, namely, all hospitalized myocardial infarctions, revascularization procedures (i.e., coronary artery bypass grafting and percutaneous interventions), heart failure, and cardiovascular deaths throughout the followup. All subjects (irrespective of residency status) were tracked nationally for vital status; death certificates were obtained from the respective states for subjects who died outside Minnesota. The underlying cause of death was coded from national mortality statistics, and cardiovascular death was defined as ICD-9 codes 390–459 and ICD-10 codes I00–I99 [9]. Systemic medication use included methotrexate, oral retinoids, cyclosporin, sulfasalazine, hydroxyurea, hydroxychloroquine, azathioprine, cyclophosphamide, leflunomide, etanercept, infliximab, adalimumab. Any use of these medications prior to baseline and during followup was considered.

Data on cardiovascular risk factors at baseline, namely, smoking, blood pressure measures, use of antihypertensive and/or lipid-lowering medications, body mass index (BMI), and diabetes mellitus, were collected as were the results of all fasting clinically performed serum lipid measures. For blood pressure, the recorded value closest to baseline ±1 year was used to calculate the risk of cardiovascular disease (CVD). For lipids, the recorded value closest to baseline ±2 years was used, since guidelines recommend measurement of lipids every 5 years.

The 10-year FRS for general CVD, which includes cardiovascular events of myocardial infarction, cardiovascular death, angina, stroke, intermittent claudication, and heart failure, was calculated [10]. For those without lipid values, we instead applied the office-based 10-year FRS which does not require laboratory values for calculation of the FRS. Since information on stroke and intermittent claudication was not collected in our study, the FRS was recalibrated to the subset of cardiovascular events collected in our population using calibration coefficients provided with the published algorithm [10].

To facilitate comparison of the predicted 10-year FRS with the observed CVD risk, the observed followup was truncated at 10 years after baseline. For patients with <10 years of followup, the predicted CVD risk was adjusted proportionately. Poisson regression models were used to obtain the standardized incidence ratio (SIR), that is, the ratio of the observed CVD in psoriasis to the FRS-predicted CVD rates. Subgroup analyses were performed by sex, age group, and exposure to systemic therapy. For the analysis of systemic therapy, patients contributed person-years of followup to the unexposed analysis until they were exposed to systemic therapy when they were moved to the systemic therapy group. An additional analysis where patients were censored at diagnosis of psoriatic arthritis was also performed.

## 3. Results

The study population included 1421 patients with incident (*n* = 480) or prevalent (*n* = 941) adult-onset psoriasis without prior CVD. Of these, 1197 (84%) patients had complete risk factor data to compute FRS, and 82 patients developed CVD during a mean followup of 8.1 years. Of the remaining 224 patients without FRS, 8 patients developed CVD during a mean followup of 7.1 years. Patients with complete information for FRS were compared to those without enough information (Table 1). For those with FRS, the predicted median 10-year FRS was 6.0% (min: 0.0%, max: 72.1%). The observed 10-year CVD risk was 6.9% (95% confidence interval (CI): 7.8%, 12%). The CVD rate was marginally lower in those without FRS (observed 10-year risk: 3.6%; 95% CI: 1.4%, 6.0%, log rank *P* = 0.084).

In 82 patients who developed CVD, 71.7 CVD events were predicted by the FRS (SIR 1.14, 95% CI: 0.92, 1.42; Table 2). The SIR was not significantly elevated for women or men. The SIR was similar in both age groups, as well as among patients using systemic therapy (Table 2). The results regarding systemic therapy remained unchanged when patients who were diagnosed with psoriatic arthritis (35 patients, 18%) were censored at diagnosis (SIR: 1.12; 95% CI: 0.62, 2.02).

## 4. Discussion

Given the mounting evidence of increased CVD risk in psoriasis, it is all more important to assess whether cardiovascular risk assessment tools such as the FRS are useful for evaluating CVD risk in these patients. This study is among the first to assess the FRS in a population-based cohort of patients with psoriasis. We found no apparent difference in the observed versus FRS-predicted 10-year CVD risk. The SIR was not significantly elevated for men or women and was similar across the age groups. This finding is in agreement with some prior studies [6, 11] and in contrast to other reports that show an excess of CVD in psoriasis [2, 12]. Some studies emphasize that the risk is greatest in younger patients (<60 years) and in patients with early-onset and longstanding psoriasis, presumably due to the fairly chronic course of the inflammatory process and cumulative disease burden perpetuating atherosclerotic disease [2, 3]. We found no apparent differences between the observed and predicted CVD risks for patients aged <60 years, which supports the findings of some investigators [13].

It has been suggested that increased cardiovascular risk in psoriasis is confined to patients with severe disease [14, 15]. Shared genetic mechanisms for the risk of psoriasis, in particular severe psoriasis, and increased cardiovascular risk have been described, including apolipoprotein E gene polymorphism [16–18]. However, severe psoriasis was found to be a risk factor for CVD in some studies [19] but not in others [11]. Using systemic therapy as a proxy for severity, we did not find apparent difference in the observed versus predicted CVD rate. This is in line with our earlier findings where systemic treatment was not significantly associated with CVD occurrence in psoriasis after adjusting for traditional cardiovascular risk factors [20]. Of note, the prevalence of severe psoriasis in population-based studies is very low [21]. Patients with severe psoriasis with increased CVD risk beyond that accounted for by the FRS likely constitute a very small percentage of patients (16% in our cohort), and this could account for the lack of difference between observed and predicted CVD risks in our study.

Strengths of this study include the longitudinal population-based design and the use of the unique REP resources. Limitations include insufficient statistical power in some subgroup analyses, in particular, analyses examining the risk among patients who received systemic treatment. These results should be interpreted with caution. Data on stroke and intermittent claudication were not available. However, calibration coefficients were applied to recalibrate the FRS to the subset of CVD events in our population which we believe minimizes this weakness. Information regarding disease severity was not available. However, we used systemic therapy as a surrogate in order to, at least in part, account for this limitation. There was no comparison cohort without psoriasis to validate the FRS accuracy in our population. However, we have previously shown that FRS accurately predicted CVD events in Olmsted County residents without rheumatoid arthritis [22]. Considering a low prevalence (<1%) of rheumatoid arthritis, we believe this analysis validates the FRS in our community. Also, other investigators using cross-sectional data have demonstrated the utility of the FRS in psoriasis [12, 23]. Finally, the population of Olmsted County, Minnesota, is predominantly White; thus, results may not be generalizable to non-White subjects.

In conclusion, the FRS reasonably estimates CVD risk in both men and women as well as in younger (<60 years) and older (≥60 years) patients with psoriasis and can be used in risk stratification in psoriasis without any further adjustment. Psoriasis did not appear to be an independent risk factor for CVD in our study. More studies are needed to better understand the nature of CVD in psoriasis and to develop effective strategies for CVD prevention in patients with psoriasis.

## Figures and Tables

**Table 1:** Characteristics and cardiovascular risk factors of patients with psoriasis and no prior cardiovascular disease. Comparison of patients with enough information to compute the Framingham risk score (FRS) to those without enough information for the FRS.

Characteristic	*n*	Patients without FRS	*n*	Patients with FRS	*P* value
Prevalent psoriasis	224	149 (67%)	1197	792 (66%)	0.92
Age, years	224	45.0 ± 12	1197	51.3 ± 13	<0.001
Male sex	224	150 (67%)	1197	565 (47%)	<0.001
Systolic blood pressure, mmHg	76	130.0 ± 13.1	1155	130.6 ± 12.7	0.78
Diastolic blood pressure, mmHg	40	78.1 ± 11.3	781	77.5 ± 11.1	0.50
Use of antihypertensive medications	224	1 (0%)	1197	225 (19%)	<0.001
Current smoking	224	38 (17%)	1197	190 (16%)	0.68
Diabetes mellitus	224	8 (4%)	1197	172 (14%)	<0.001
Body mass index, kg/m^2^	0	—	1080	28.6 ± 6.4	—
Use of lipid-lowering medications	224	0 (0%)	1197	125 (12%)	<0.001
Any systemic treatment	224	36 (16%)	1197	162 (14%)	0.31
Total cholesterol, mg/dL	36	220.3 ± 40.8	968	205.7 ± 35.1	0.032
LDL, mg/dL	34	138.0 ± 39.0	957	120.3 ± 34.8	0.005
HDL, mg/dL	34	46.2 ± 7.1	958	49.9 ± 8.3	0.019
Triglyceride values, mg/dL	15	196.9 ± 96.9	688	165.9 ± 110.2	0.11

Values in the table are *n* (%) or mean ± SD.

LDL: low density lipoprotein; HDL: high density lipoprotein; SD: standard deviation.

**Table 2:** Comparison of observed and FRS-predicted CVD risk in patients with psoriasis.

Group	Number of patients	CVD events		SIR (95% CI)
		Observed	FRS-predicted
Total	1197	82	71.7	1.14 (0.92, 1.42)
Female	632	33	25.7	1.28 (0.92, 1.79)
Male	565	49	45.9	1.07 (0.81, 1.41)
Age				
<60 years	879	34	33.5	1.01 (0.73, 1.41)
60+ years	318	48	38.1	1.26 (0.95, 1.68)
No systemic treatment	1364	71	61.8	1.15 (0.91, 1.45)
Any systemic treatment	198	11	9.8	1.12 (0.62, 2.03)

FRS: Framingham risk score; CVD: cardiovascular disease; SIR: standardized incident ratio; CI: confidence interval.
